# Electronic Waste Governance under “One Country, Two Systems”: Hong Kong and Mainland China

**DOI:** 10.3390/ijerph15112347

**Published:** 2018-10-24

**Authors:** Natalie W. M. Wong

**Affiliations:** Department of Public Policy, City University of Hong Kong, Hong Kong, China; nataliew@cityu.edu.hk; Tel.: +852-3442-4596

**Keywords:** electronic waste, Hong Kong, China, waste control, policy network, zero waste

## Abstract

China is one of the largest e-waste dumping sites in the world, and Hong Kong, a semi-autonomous territory in China, is also affected by illegal e-waste disposal and transfer. While the Chinese government implemented a waste import ban in January 2018, Hong Kong has not enforced Chinese policies under the “One Country, Two Systems” framework. Drawing on a policy network approach, this paper provides an explanatory framework for e-waste governance in Hong Kong and China, and identifies the major obstacles to shaping effective transboundary e-waste control and prevention. The paper argues that institutional arrangements play a dominant role in governing e-waste policy networks at the local level of governance in Hong Kong and China; however, a lack of accountability and capacity at the transboundary level can explain the different waste electrical and electronic equipment (WEEE) management strategies in these two places.

## 1. Introduction

### 1.1. Study Background

**Figure 1:**
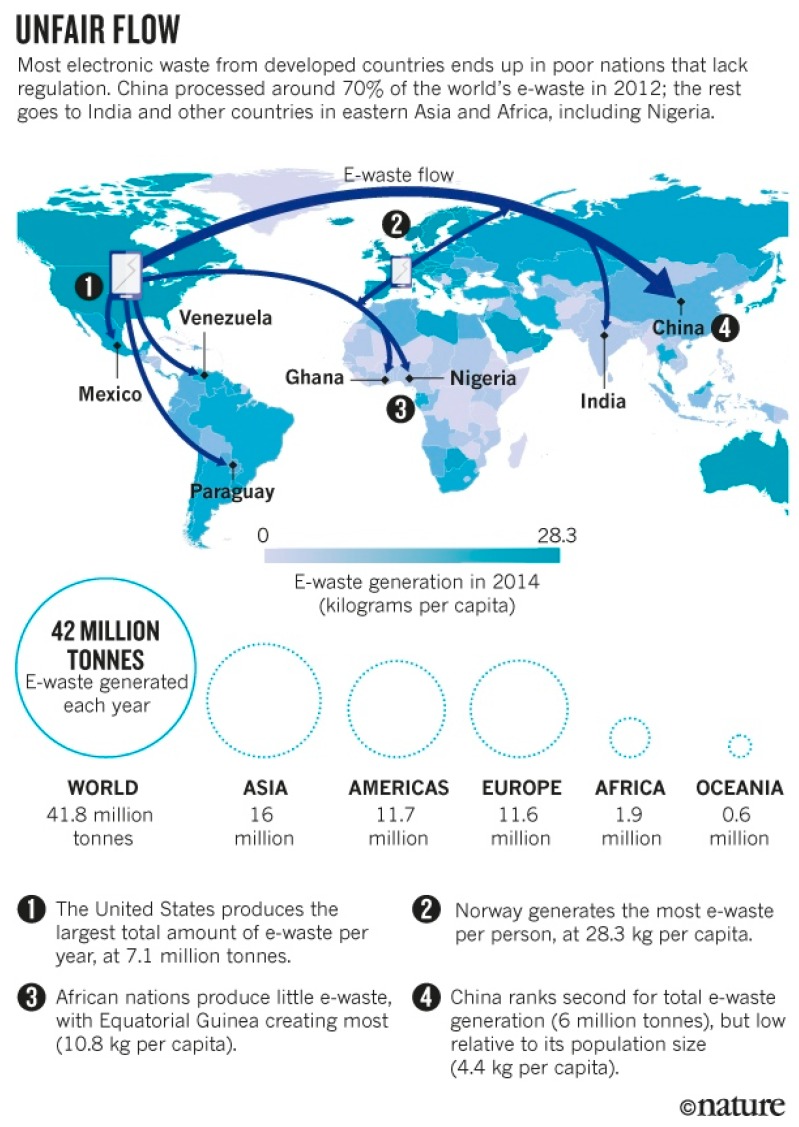
Transboundary movement of waste electrical and electronic equipment (WEEE) [9].

Electronic devices are continuously being improved and developed, and the stream of new, faster, smarter, and better gadgets leads to increases in electronic consumption in this fast-changing digital environment. Waste electrical and electronic equipment (WEEE), however, is the outcome of this environment. The United Nations Environment Programme highlighted that electronic waste is subsequently increasing; more than 44.7 million metric tonnes of electronic waste (e-waste) was generated globally in 2016, representing a threat to the environment [1]. WEEE generally refers to discarded electronic goods or appliances, such as computers, smart phones, refrigerators, and washing machines [2]. The neoliberal trade in the globalized world and the transboundary movement of electronic waste has led to negative impacts on the environment and human health. Valuable materials such as copper and gold have become a major source of income to developing countries, and studies have found that the trade of electronic waste occurs from developed countries to developing countries with lax regulations, such as China [3]. Seventy percent of electronic waste ends up in China eventually owing to this transboundary e-waste movement (Figure 1), making this country the largest e-waste dumpsite in the world [3]. A previous investigation found that Guiyu, a small town in the southeastern part of China, is a major hub for dismantling electronic waste, where the workers and villagers suffer from illness due to toxicity and the land is polluted [4]. In addition, 350 million tonnes of electronic waste have been imported to China from different part of the world in the last four decades [5]. In light of this information, the Chinese government suspended the import of electronic waste in 2000 [6]. The Chinese government signed the Basel Convention, an international convention for suspending transnational hazardous waste movement, in 1990, which took effective in 1992. Additionally, the central government implemented regulations to ban import waste in 2018 [7]. Meanwhile, Hong Kong, a former British colony that was returned to China with partial autonomy in 1997, has also faced the growth of imported electronic waste in the last few decades. Basel Action Network (BAN), an environmental group, estimated that 1000 tonnes of electronic waste have been imported to Hong Kong every day from different parts of the world [8]; the group believes that a portion of this amount of electronic waste is transported to other cities in China illegally [8].

The Hong Kong government has made efforts to suspend the import of electronic waste and applied the Basel Convention under the framework of “One Country, Two Systems” [10]; in addition, regulations are promulgated to control the growth of electronic waste [11]. However, legal loopholes concerning the transboundary movement of electronic waste remain. Although the Chinese government bans the import of foreign waste, Hong Kong has allowed licensed imports and may serve as an entrepot, exporting waste to other countries or moving it into China [8]. The Chinese government further banned all foreign import waste, which led to this waste ending up in Hong Kong. The New Territories, located in the north of Hong Kong, is the main dumping site for electronic waste [8]. The illegal disposal of discarded electrical devices not only pollutes the surround area but also has potential risks in the long run. In controlling and managing e-waste disposal, both Chinese and Hong Kong authorities have designed different measures for e-waste avoidance in line with the concept of zero waste to achieve the goal of a sustainable society.

Both the Hong Kong and Chinese governments have implemented various strategies to control the import of WEEE; however, the outcomes have been different within each territory. Under the framework of “One Country, Two Systems”, Hong Kong maintained capitalist economic and political systems after the handover on 1 July 1997. Thus, the two governments adopt different policies in environmental management. Against this background, this research study focuses on the variance in e-waste governance between Hong Kong and China, which has led to a lack of law enforcement in e-waste avoidance. In our investigation, we pose the following research questions: (1) How has the e-waste governance shaped policy implementation in Hong Kong and China? (2) Why do Hong Kong and Chinese authorities implement policies regarding e-waste avoidance differently? This study aims to explore the explanatory models in e-waste governance between Hong Kong and China to explain why the authorities control e-waste differently within a single country.

Hence, this study seeks to understand how e-waste governance is shaped by broader factors, such as institutional factors, disposal capacity, and features of e-waste generation and composition. After that, the policy network approach is employed to explain how the agencies of each government have shaped the outcomes of e-waste avoidance differently, and to address how different strategies have affected the authorities’ control over the import of e-waste. This study adopts secondary materials including, for example, governmental reports and data on waste management systems and the disposal of electrical appliances, to understand governance differences between Hong Kong and China. This study shows how the different political systems between Hong Kong and China have affected e-waste avoidance in the global neoliberal trade system. The research explores patterns of e-waste generation and disposal in China and Hong Kong, identifies the multiple agencies controlling the e-waste in these two places, and addresses the transboundary movement of e-waste and the gap of law enforcement. In addition, the research provides an explanatory framework for understanding the strategy of transboundary e-waste management between Hong Kong and China.

### 1.2. Policy Networks for Exploring the Models of WEEE Management

The policy network model shows the interdependent relations within the policy-making process and reveals how different actors form the networks in a policy field. The actors, mainly sub-national agencies, are interdependent in that they achieve their goals by exchanging their resources. Thus, the actors form networks to achieve policy goals. The actors, however, have their own goals and strategies, while the policy is the result of bargaining [12,13]. At the same time, their actions are constrained by their rules [14]. Nevertheless, an institutional context is important to the formation of policy networks because the interactions of actors are often linked in organizational arrangements [12,14]. Thus, it is necessary to explain the factors affecting the emergence of policy networks.

The institutional arrangements here focus on the preconditions for the rise of policy networks, such as the extent to which the state encourages the formation of policy networks. As such, the state has close ties to institutional arrangements in providing opportunity for the emergence of policy networks. The perception of equal relationships among actors in networks is being challenged because policy outcomes may be altered by the role of the state, the network membership, and the preferences of participants. As such, policy networks are regarded as “organized entities” of a “division of labor” because different tasks are distributed among actors to coordinate and guide the policy outcome [15].

### 1.3. Previous Related Studies

#### 1.3.1. Previous Studies on E-Waste Impacts and Management

Previous studies on e-waste governance have mainly focused on national and international levels. At the national level, the literature has discussed the politics of managing e-waste among different state and non-state actors [16,17]. Meanwhile, at the international level, the e-waste governance between developed and developing countries is studied addressed the environmental injustices occurring under economic globalization are addressed [18]. The discussion on e-waste in both Hong Kong and China, however, has mainly focused on the environmental impacts in both places, including the generation of e-waste and its management [19,20]. The environmental advocacy group Basel Action Network (BAN) had investigated the flow of illegal cross-boundary e-waste trading between Hong Kong and China. However, all these studies have not studied the governance of e-waste. Hence, this study provides a view of e-waste governance on one hand, and the interaction between the agencies to explain the weakness of e-waste control on the other hand.

#### 1.3.2. Transboundary Environmental Collaboration between Hong Kong and China

Transboundary environmental cooperation between Hong Kong and Mainland China have addressed environmental practitioners and researchers since the Sino-British Joint Declaration was signed in 1984 and since the handover in 1997. Currently, the governments have initiated cooperative efforts, for instance a Joint Working Group on Sustainable Development and Environmental Protection and a Joint Working Group on Cleaner Production between Hong Kong and Guangdong Province, to tackle regional environmental problems.

Geographically, Hong Kong has a close link to Guangdong Province through megacities such as Shenzhen, Guangzhou, and Dongguan, which explains the occurrence of transboundary environmental challenges commonly faced by the Hong Kong and Guangdong provincial governments. Currently, both governments have signed joint agreements, such as the Cooperation Agreement between Hong Kong and Guangdong on Combating Climate Change (2011), the Pearl River Delta Regional Air Quality Management (2003), and the Deep Bay (Shenzhen Bay) Water Pollution Control Joint Implementation Programme (2000). In addition, both governments have established collaborative mechanisms for managing transboundary environmental issues, for instance the Joint Working Group on Sustainable Development and Environmental Protection (1998). This Joint Working Group has covered different issues on environmental protection and sustainable development, such as air and water qualities, marine conservation, and town planning. What is more, both authorities have signed the Hong Kong-Guangdong Cooperation Agreement on Cleaner Production, which aims to promote energy efficiency and emission reduction. Recalling the concept of interagency collaboration at the transboundary level, both Hong Kong and Mainland China authorities have mainly engaged in agreement and coordination [21] to deliver joint services [22]. However, transboundary cooperation to tackle the movement of electronic waste between Hong Kong and Mainland China has been in vain.

**Figure 2:**
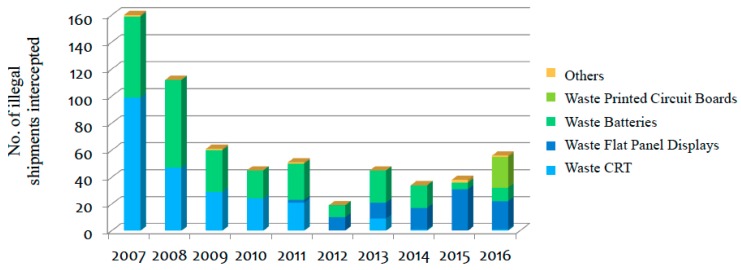
Number of illegal shipment intercepted in 2007–2016 [29].

Previous studies on transboundary environmental cooperation between Hong Kong and Mainland China, however, have pointed out that institutional constraint is a hindrance to the cooperation [23,24,25,26], which also demonstrated in this study. Although both Hong Kong and Chinese authorities have separately implemented different measures on controlling the import, export, and transit of hazardous waste including WEEE, as well as signed the Cooperation on Control of Waste Movements Between the Mainland and HKSAR, investigations have found that the transboundary movement of WEEE between Hong Kong and China is still significant. The Basel Action Network, an environmental non-governmental organization advocating the suspension of transnational hazardous waste movement, traced American recyclers and discovered that the toxic electronic equipment had been exported to China via Hong Kong [27]. The illegal transportation of toxic waste was found in Hong Kong and the original exporters were developed countries, such as the United States, Canada, Japan, and countries in the European Union (Figure 2). For example, the Environmental Protection Department of Hong Kong seized 760 tonnes of e-waste in 2010—discarded electronic equipment including notebook computer batteries, CRT, and waste ray tubes—and the shipments planned to go China [28], p. 15. In addition, second-hand electrical equipment was exported to Hong Kong and transferred to China. For example, 2.83 million second-hand TV sets, 1.35 million computer monitors, and 541,000 second-hand air conditioners were exported to China via Hong Kong in 2005 and 2006 [28], p. 15.

The Chinese government adopted measures to suspend the import of toxic waste in 2000, but the legislative difference between Hong Kong and China provided a loophole in controlling WEEE. Under the “One Country, Two Systems”, Hong Kong implements separate regulations on controlling transboundary toxic waste movement. However, it is legal for Hong Kong to import or act as an entrepot for second-hand EEE and e-waste if an import license is obtained in Hong Kong. Moreover, the equipment imported to Hong Kong can be shipped to other countries, including China, with no waste permit required from Hong Kong authorities. As such, the different measures and regulations between Hong Kong and China provide loopholes in controlling transboundary WEEE, meaning that toxic waste can still be easily shipped to China. Although the Chinese government has implemented strict regulations to ban all waste, including WEEE, from other countries since January 2018, electrical equipment still ends up in dumping sites in Hong Kong [7]. The loopholes in transferring WEEE not only show the different legislation between Hong Kong and China but also reveal the lack of coordination and lack of law enforcement by the authorities. Hence, the explanatory frameworks of managing WEEE in Hong Kong and China are elaborated in the next section in order to explain the correlations between institutional arrangements and different actors in managing WEEE in Hong Kong and Mainland China. This study explores the governing models of e-waste in Hong Kong and China, in addition to addressing the different political systems that explain the gaps in electronic waste management.

## 2. Methodology and Case Studies

To gather information and data to map the generation and disposal of e-waste in Hong Kong and China, as well as identify governance agencies and mechanisms, an extensive literature review on waste management systems was carried out through scholarly books, peer-reviewed journal papers, white papers, reports, and various online sources. The findings from the literature review on e-waste management systems indicate that traditional e-waste waste management includes generation, collection, treatment, and disposal. Building on these materials, causal correlations are further developed into explanatory frameworks to examine e-waste governance between China and Hong Kong.

### 2.1. E-Waste Governance: An Explanatory Framework

In this section, factors that shape e-waste governance are unpacked and casual correlations are developed into an explanatory framework.

The dependent variables are

(i) e-waste disposal and treatment;
(ii) e-waste generation.

The independent variable is

(i) involvement of actors (e.g., public and private actors).

The control independent variable is

(i) institutional arrangements for e-waste management.

This study deploys policy networks to examine e-waste governance in both Hong Kong and China. The study includes literature on the connections between local agencies and governments in governance processes. In e-waste governance, the institutional arrangements have incorporated cross-levels of governance as well as networks of actors. These arrangements span local as well as transboundary levels. Exploring these structures in Hong Kong and China and their jurisdictions, the following hypothesis is generated:

The degree of departmental coordination affects WEEE control.

The degree of departmental coordination refers to the extent to which the agencies share information with each other to reduce and control the disposal and treatment of WEEE. Additionally, the institutional arrangements for e-waste management have shaped the powers and responsibilities of the agencies, as well as the involvement of actors. Two levels of governance are examined: the local level of governance in Hong Kong and China, and the transboundary level of governance between Hong Kong and China.

### 2.2. A Case Study of E-Waste Management in Hong Kong and China

#### 2.2.1. E-Waste Generation and Composition

According to the Environmental Protection Department (EPD) of Hong Kong, 70,000 tonnes of discarded computers and electrical and electronic equipment are disposed every year [30].

**Figure 3:**
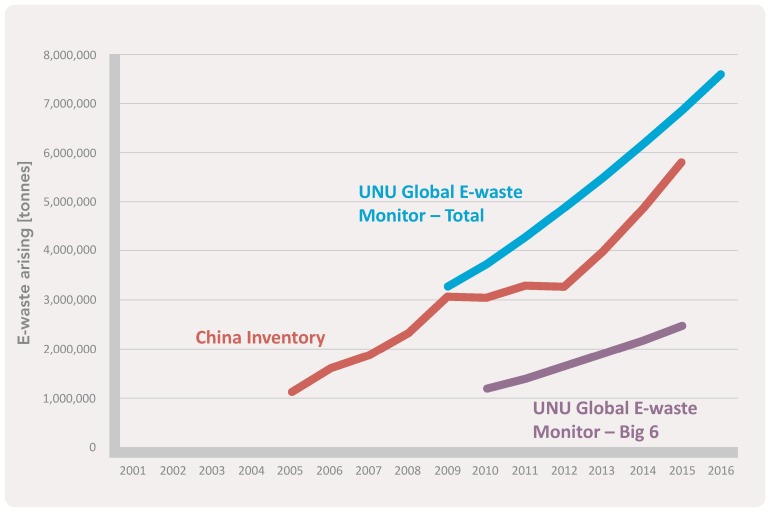
E-waste arising in China [31], p. 143.

Before the Chinese government implemented regulations to suspend foreign waste in January 2018, the sources of WEEE in China included both imports and domestic generation (Figure 3). Annually, there are 500 million tonnes and 200 million tonnes of WEEE imported and generated by the domestic market, respectively [28], p. 12. Five major electronic appliances (TV sets, refrigerators, washing machines, air conditioners, and computers) constitute the major sources of WEEE import and domestic generation. With the subsequent growth of WEEE, China has turned into a global WEEE dumping site [3].

#### 2.2.2. E-Waste Collection and Recycling

**Figure 4:**
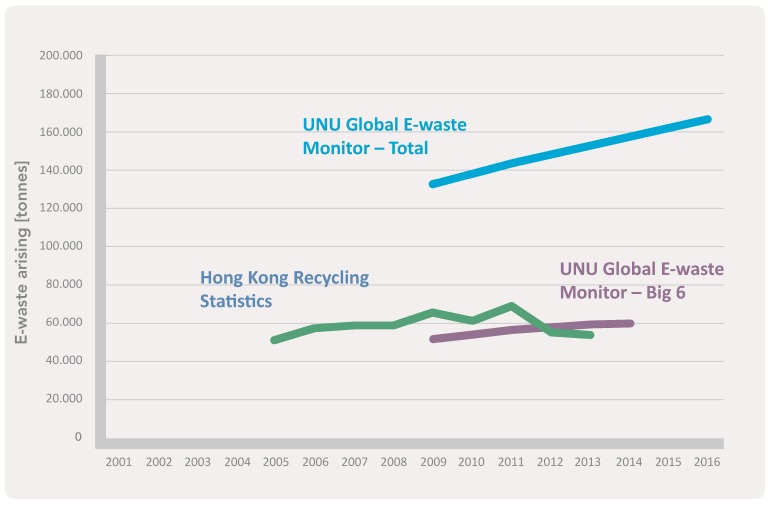
Number of recycled WEEE in Hong Kong between 2004 and 2014 [31], p. 130.

In Hong Kong, there are fewer than 70,000 tonnes of WEEE recycled during 2005–2013 (Figure 4) and unidentified amounts of discarded WEEE are sent to landfills [30], which leads to negative impacts on the environment. Starting in 2003, the Environmental Protection Department implemented various measures for collecting discarded WEEE. The EPD introduced the Territory-Wide Trial Recovery Programme with two non-profit organizations in 2003 to collect and recover discarded WEEE. The program has successfully received more than 40,000 pieces of WEEE each year [30] and these refurbished computers and electrical appliances are donated to the needy, while those beyond repair are sold to recyclers [30]. Two years later, the authority launched the Waste Electrical and Electronic Equipment Recycling Programme to recover discarded WEEE instead of disposing it. With financial support from the Environmental and Conservation Fund (ECF) and founded by the HKSAR government, the St. James Settlement also collected WEEE for recycling or reuse. The collected equipment was again either recycled or donated to families in need [30]. The EPD also sub-contracted an operator, ALBA Integrated Waste Solution (ALBA-IWS), under the Progressive Implementation of Producer Responsibility Scheme in 2016 to collect and dismantle WEEE [30]. In addition, the authority regulated eight types of electrical equipment (i.e., air-conditioners, refrigerators, washing machines, televisions, computers (including desktops, laptops, and tablets), printers, scanners, and monitors) in this scheme, and suppliers and sellers are requested to provide free removal services and send abandoned equipment to downstream recyclers for proper treatment [32]. Moreover, the EPD established e-waste collection vehicle stations in 18 administrative districts to collect e-waste [33].

Recycling companies also receive abandoned electrical equipment, mainly from informal waste pickers. These companies are usually small-scale outlets and collect recyclable appliances from informal waste pickers at very low prices. After receiving the electrical appliances, these recycling companies usually dismantle the discarded appliances to extract the metals, which are sold to factories or exported to other countries.

Both formal and informal collectors engage in WEEE collection in China. Informal WEEE collectors, similar to waste pickers in Hong Kong, are self-employed and migrant workers who usually originate from rural areas and travel around in urban cities to purchase discarded appliances and sell them in second-hand markets or to downstream recyclers [28], p. 14. Investigations have indicated that a town in Guiyu of Southern China is the biggest hub of WEEE sites in the country. Hundreds of downstream WEEE recyclers are found in the town, and it is estimated that thousands of villagers are employed in the town for dismantling WEEE [2]. These investigations also highlighted the problems of informal recyclers in Guiyu, as they disassemble discarded electrical appliances without proper tools or facilities—not only polluting the surrounding environment but also bringing negative impacts on human health. The Medical University of Shantou reported that 165 blood samples among the children in Guiyu exhibited a high level of contaminants, such as lead [2]. However, the Chinese government has not expressed any intention to ban informal WEEE collection [28], p. 17.

The Law of the People’s Republic of China on the Prevention and Control of Environmental Pollution by Solid Waste was implemented in 2005 to institutionalize municipal solid waste management, including WEEE. However, the law has not had any arrangement for building waste collection mechanisms, and waste collection mainly relies on recycling companies. Currently, recycling companies are required to have a treatment license for engaging in the recycling, dismantling, and disposal of WEEE, which is issued by the Ministry of Environment. Compared to the aforementioned downstream recyclers, these formal collectors are large-scale and state-owned businesses. There are more than 100,000 recycling companies in the country and these companies are integrated recyclers, so WEEE is part of their business [34].

#### 2.2.3. E-Waste Treatment and Disposal

**Figure 5:**
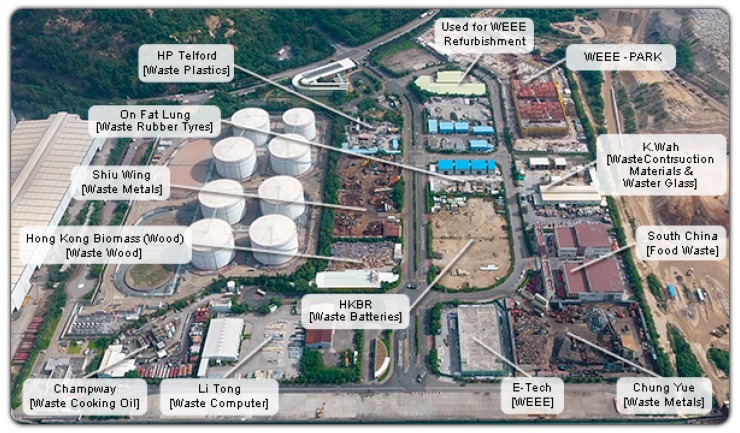
Eco Park, Hong Kong [35].

Before the Hong Kong government implemented the Progressive Implementation of Producer Responsibility Scheme, discarded equipment was to send to landfills with other municipal solid waste or sold to downstream recycling companies. However, the authority engaged with a non-profit organization and set up the Kowloon Bay Waste Recycling Centre for collecting and recycling e-waste and municipal solid waste; later, in the Policy Framework for the Management of Municipal Solid Waste (2005–2014), published in 2005, the government set out a strategy for waste management that placed emphasis on waste reduction and recovery. Furthermore, the EcoPark WEEE Recycling Centre opened in 2011 for recycling used electrical and electronic appliances (Figure 5).

EcoPark is the first recycling business park in Hong Kong and provides a rentable area of 140,000 m^2^ long-term land at affordable costs for the recycling industry. Currently, 13 companies rent the land in the park, and two of them recycle discarded electrical equipment and computer waste [36]. The center dismantles and recycles the used electronic equipment and donates equipment to the needy. In addition, the recently implemented Progressive Implementation of Producer Responsibility Scheme details the subcontracting of WEEE recycling services to ALBA-IWS. ALBA-IWS has a capacity to handle 30,000 tonnes of e-waste annually, turning it into resources after a series of detoxification, dismantling, and recycling processes [30].

According to the Ministry of Commerce of People’s Republic of China, the largest recycling companies in the country (for example, Gezhouba Group and GEM Co. Ltd.) recycle not only WEEE but also other municipal solid waste [34]. These companies have set up recycling chains for collecting, processing, and remanufacturing waste, ultimately turning it into vulnerable resources. For example, GEM Co. Ltd. has set up seven WEEE processing centers in Hubei, Jiangxi, Henan, Jiangsu, Shanxi, Inner Mongolia, and Guizhou and recycled 1.2 million tonnes of WEEE annually [37]. Furthermore, the Chinese government encourages recycling work to be combined with waste separation. For example, Beijing Huangwei (Beijing Environment and Hygiene Group), a Beijing-based state-owned enterprise, combines both waste separation and recycling, including WEEE, for better effectiveness in waste management in the city of Beijing [38].

#### 2.2.4. Regulatory Policies for E-Waste

The Hong Kong government signed the Basel Convention in 1989, but it has not implemented strict regulations for WEEE disposal. The Waste Disposal (Chemical Waste) (General) Regulation has been the only regulation for WEEE disposal, and has set a permit system for controlling the import and export of toxic waste in line with the requirements of the Basel Convention. The authority implemented a clear target for sustainable development until 2013. The Environment Bureau, the agency for implementing environmental policy, issued the Policy Framework for the Management of Municipal Solid Waste (2005–2014) and the Hong Kong Blueprint for Sustainable Use of Resources (2013–2022), aiming to reduce the volume of municipal solid waste disposal rate by 40% on a per capita basis by 2022 [39], and WEEE is part of the action plan in the Blueprint. The Bureau promulgated the Producer Responsibility Scheme on WEEE in 2018 and further regulations on WEEE will be put into effect on 1 August 2018 [40]. Under the new regulations, suppliers of air-conditioners, refrigerators, washing machines, televisions, computers, printers, scanners, and monitors, collectively referred to as regulated electrical equipment (REE), are required to be registered with the EPD (Table 1). Moreover, the suppliers are required to pay recycling levies as well as provide recycling labels when distributing regulated electrical equipment. At the same time, the sellers must have a removal service plan endorsed by the department in order to sell the regulated electrical equipment [40] (Table 2).

The Chinese government has implemented various measures for WEEE management, as well as to control WEEE import, since the signing of the Basel Convention in 1992. Starting in 2000, the authority issued the Catalogue for Managing the Import of Waste, aiming to suspend the import of e-waste; they also later implemented the Technical Policy on Pollution Prevention and Control of WEEE (2006) to set the principles of “3R” (reuse, recycle, reduce), as well as the provision for environmental friendly collection, reuse, recycling, and disposal of WEEE. The authorities further institutionalized WEEE management and promulgated the Ordinance on Management of Prevention and Control of Pollution from Electronic and Information Products (2007) to set the standards for eco-friendly production design and restrictions on the use of hazardous substances. The Administrative Measures on Pollution Prevention of WEEE in 2008 aimed to prevent any pollution caused by the disassembly, recycling, and disposal of e-waste. Additionally, the government issued the license scheme for WEEE recycling companies. In the Regulation on Management of the Recycling and Disposal of Waste Electrical and Electronic Equipment (2011), the government made WEEE recycling mandatory and implemented extended producer responsibility, in addition to setting up a fund to support WEEE recycling (Table 3). Finally, the strict regulation suspending all import waste, including WEEE, took effective in January 2018, representing an even further step taken by the government to control the import of waste (Table 4).

## 3. Results and Discussion

The case studies of implemented practices in Hong Kong and Mainland China, described in Section 2.2, refer to WEEE generation, collection, and disposal, treatment facilities, and regulatory policies for WEEE management. In the case of Hong Kong and China, the information presented in Section 2.2 refers to the hypothesis stated in Section 2.1. It was found that the coordination among governmental agencies affects WEEE management, but is manipulated by the institutional arrangements.

In the case of Hong Kong, both the Customs and Excise Department and the Environment Bureau are the main authorities for managing WEEE and implemented regulations on controlling discarded electric equipment. The former is responsible for controlling the illegal import of electronic equipment, while the latter, along with its subordinate department, the Environmental Protection Department, is responsible not only for setting regulations on controlling WEEE disposal and treatment, but also for establishing WEEE collection and recycling treatment facilities. Notably, the Environmental Protection Department collaborates with the Customs and Excise Department to conduct spot-checks of waste consignments at import and export control points for the prevention of illegal WEEE shipments [41]. In building WEEE treatment and recycling facilities, the Environmental Protection Department has subcontracted this work out to private companies and local environmental groups [42].

The Ministry of Ecology and Environment and the General Administration of Customs are the major agencies controlling WEEE in China. The General Administration of Customs controls the illegal import of discarded electronic appliances and collaborates with three other authorities: the Ministry of Ecology and Environment, the Ministry of Public Security, and the General Administration of Quality Supervision, Inspection, and Quarantine [7]. The Ministry of Ecology and Environment carries out measures to manage WEEE treatment and disposal, such as implementing the suspension of foreign waste import. Gaining support from the Ministry of Ecology and Environment, private corporations receive regular financial subsidies for WEEE disassembly and material recovery [43]. In this sense, the local institutional arrangements in Hong Kong and Mainland China define their responsibilities in managing WEEE, and the environmental protection authorities have engaged the involvement of non-governmental actors in managing WEEE.

However, the local level of policy networks does not explain the transboundary WEEE movement between Hong Kong and Mainland China. As mentioned in the previous section, the Chinese government signed the Basel Convention and Hong Kong will follow the same footsteps to control the import of WEEE. The different environmental managements under the framework of “One Country, Two Systems” has enabled loopholes to be exploited in the management of WEEE between Hong Kong and China. Hong Kong allows licensed imports and can serve as an entrepot for shipments entering China or other countries. The transboundary mechanism requires environmental collaboration, for example air pollution, but the different jurisdictions between Hong Kong and China cannot solve the abovementioned loophole. This loophole further reveals the missing link between the two policy networks. Policy networks involve the delegation of power according to four features: legitimacy (the authority is acknowledged the right to rule), accountability (to account for the consequence of exercising the power), capacity (the acquisition of resources to exercise the power), and enforcement (carrying out the power) [44]. Given this, transboundary policy networks should have a mechanism involving all four of these characteristics. According to our review of the existing transboundary environmental collaboration mechanism, it is a communication platform for exchanging environmental information without any accountability or enforcement to ensure proper WEEE management. Moreover, there is a lack of consensus between the two authorities concerning the definition of discarded electronic appliances. Together, these factors explain why WEEE management is treated differently between Hong Kong and China.

## 4. Conclusions

China is one of the major discarded electronic appliance hubs in the world, and Hong Kong, a semi-autonomous city in the southern part of China, acts as a main entrepot for WEEE exports to different countries, including China. Given this, the purpose of this study was to discuss the control and prevention of WEEE in Hong Kong and Mainland China. Employing the policy network approach, we developed an explanatory framework to examine WEEE management at both the local and transboundary levels in Hong Kong and Mainland China. It was found that the institutional arrangements laid out the WEEE management systems in both places. In governing the policy networks of the WEEE spectrum, both environmental authorities in Hong Kong and China play a dominant role. However, the status of free trade port in Hong Kong and weak law enforcement in Mainland China explain the continued import of discarded electronic appliances. Furthermore, the different environmental jurisdictions and poor implementation policy capacities in the transboundary environmental collaboration mechanism between Hong Kong and Mainland China also explains the different levels of WEEE control, which must be improved in order to move towards a zero-waste society.

## Figures and Tables

**Table 1:** Stakeholders involve in WEEE governance in Hong Kong.

Stakeholders Involve in WEEE Governance in Hong Kong
Legislative Council
Environmental Protection Department
Private recyclers
Environmental groups
Hong Kong WEEE Recycling Association
Consumers
Customs

**Table 2:** Summary of WEEE management framework in Hong Kong.

**Legal Framework**	No specific e-waste law exists in Hong Kong; however, the Promotion of Recycling and Proper Disposal (Electrical Equipment and Electronic Equipment) (Amendment) Bill, 2015 (PRS Scheme) was under discussion at the time of writing.
	The Waste Disposal Ordinance (WDO) (1980), commonly known as the WDO, controls and regulates storage, collection, and disposal, including the treatment, reprocessing, and recycling of waste.
	Under the WDO, import and export of hazardous wastes, including e-waste is subject to permit control.
**Collection Mechanism**	Under the WEEE Recycling Program and Computer Recycling Program, 16 public collection points and mobile collection vehicles have been designated by the EPD to collect WEEE from different districts.
	EPD’s mobile collection vehicle collects electrical appliances, as well as computers, rechargeable batteries, compact fluorescent lamps, and fluorescent tubes. The vehicle visits a different district each week.
	The Computer Recycling Programme Free collection service is provided to the public, on special request, for bulk pick-up (five or more pieces of main computer equipment—i.e., desktops, notebooks, printers, scanners, and CRT & LCD monitors)
	Under the new contract signed by the government, the recycling contractor will set up eight collection points and three recycling centers across the city.
**Processing Infrastructure**	According to the EPD, almost 80% of e-waste is exported to Mainland China and other countries for recycling. The rest is either dumped in one of the three operating strategic landfills or temporarily stored in open storage sites in rural New Territories or sent to local recycling facilities. Only 10% of e-waste generated is currently recycled locally.
	Alba Integrated Waste Solutions Hong Kong, a joint-venture subsidiary of the Alba Group, signed a 12-year contract with the Hong Kong government in May 2015. It will spend two years building the plant and then operate the collection and recycling system in the city for the next 10 years. The plant would be capable of processing 30,000 tonnes of waste a year, but the capability could be extended to a maximum of 56,000 tonnes by arranging additional shifts as needed.
**EHS Standards**	Local collectors, refurbishers, and recyclers are subject to compliance of environmental standards, random audits/patrolling by EPD and are required to submit reports in accordance with licensing conditions.
	Pollutants produced in workshops are subject to control under Air Pollution Control, Noise Pollution, Water Pollution Control, and Waste Disposal Ordinances.

**Table 3:** Stakeholders involve in WEEE governance in Mainland China.

Stakeholders Involve in WEEE Governance in the Mainland China
National Development and Reform Commission
Ministry of Ecology and Environment
Ministry of Commerce and Ministry of Finance
Customs
Environmental Groups
Informal recycling collectors

**Table 4:** Summary of WEEE management framework in Mainland China [32], p. 143.

**Legal Framework**	China has ratified both the Basel Convention and the Ban Amendment; however, it struggles with huge quantities of e-waste imports. Important laws related to e-waste management in China are as follows: (1) the Law on the Prevention and Control of Environmental Pollution by Solid Wastes was passed in 1995 and amended in 2005; (2) the Catalogue for managing the import of wastes (MOC, MEP, NDRC, GAC, AQSIQ, 2009, No. 36) has banned the import of e-waste since 2000; (3) the Technical Policy on Pollution Prevention and Control of WEEE (SEPA No. 115) came into force in 2006 and sets other “3R” and “Polluter Pays” principles, stipulates eco-design, and makes provisions for environmentally sound collection, reuse, recycling, and disposal of WEEE; (4) the Ordinance on Management of Prevention and Control of Pollution from Electronic and Information Products, commonly known as China RoHS (MIIT No. 39), has been in force since 2007. It sets requirements for eco-design, restrictions on use of hazardous substances and requirements for producers to provide information about their products. (5) Since 2008, the Administrative measures on pollution prevention of WEEE (SEPA No. 40) has focused on preventing pollution during disassembly, recycling and disposal of e-waste and has provided a licensing scheme for e-waste recycling companies. (6) Regulations on the Management of the Recovery and Treatment of Waste Electronic and Electrical Products, commonly known as China WEEE Regulation, was passed in 2009 and came into force in 2011. It makes e-waste recycling mandatory, implements EPR, and establishes a fund to subsidize e-waste recycling. The first batch of products covered under this law was limited to TVs, refrigerators, washing machines, air conditioners, and computers. In the second batch, this catalogue will be expanded to printers, copiers, mobile phones, water heaters, and monitors, among others. The administrative measures for levy and use of treatment fund for WEEE includes a levy on EEE is used to fund the collection and treatment of WEEE. Other related legislations are as follows: (1) the Cleaner Production Promotion Law, passed in 2002 and amended in 2012; (2) the Circular Economy Promotion Law, passed in 2008.
**Collection Mechanism**	Most of the e-waste is collected by the informal sector collectors who offer door-to-door collection services and make cash payments to purchase e-waste from households and businesses.
**Processing Infrastructure**	Formal treatment infrastructure: Currently, 130 e-waste recycling enterprises are registered on the e-waste Dismantling Enterprise list, and as of 2012, 53 e-waste treatment facilities in 15 provinces and cities had received the necessary treatment licenses, with a total of 122 planned to be built by 2015 (MEP). However, formal treatment is still in early stages and most of the e-waste is recycled informally.
**EHS Standard**	Municipal environmental protection departments are responsible for approving the qualifications of enterprises engaged in WEEE treatment, based on the requirements set down under the WEEE Treatment Facility Qualification. The informal dismantling and recycling sector does not ensure safe e-waste practices, and it has caused extreme environmental degradation and increased health risks to those involved in such recycling activities. Studies have shown that residents of places that are recipients of e-waste in China that are also hubs of informal waste activities are exposed to dioxins 15–20 times higher than the WTO recommended level.

## References

[1] Kennedy J. (2015). Illegal Trade in Toxic E-Waste to Rise Sharply: Study.

[2] Waste Electrical & Electronic Equipment (WEEE). http://ec.europa.eu/environment/waste/weee/index_en.htm.

[3] China: The Electronic Wastebasket of the World. https://edition.cnn.com/2013/05/30/world/asia/china-electronic-waste-e-waste/index.html.

[4] Greenpeace (2009). Guiyu: An E-Waste Nightmare.

[5] China Officially Issued a Ban on “Foreign Garbage” in 2018, Hong Kong’s Garbage Recycling Industry Is about to Transform. http://huanbao.ne21.com/show-1028.html.

[6] Database of Laws and Regulations (2007). Law of the People’s Republic of China on the Prevention and Control of Environmental Pollution by Solid Waste.

[7] General Office of the State Council (2007). Notice of the General Office of the State Council on Issuing the Implementation Plan for Prohibiting the Entry of Foreign Garbage and Advancing the Reform of the Solid Waste Import Administration System.

[8] U.S. Environmental Group Conducted Transnational Investigation, Hong Kong Turns to Global E-Waste Dumpsite. https://www.hk01.com/%E7%A4%BE%E6%9C%83%E6%96%B0%E8%81%9E/22766/01%E7%8D%A8%E5%AE%B6-%E9%A6%99%E6%B8%AF01-%E7%BE%8E%E5%9C%8B%E7%92%B0%E5%9C%98%E8%B7%A8%E5%9C%8B%E8%AA%BF%E6%9F%A5-%E6%B8%AF%E6%B7%AA%E5%85%A8%E7%90%83%E9%9B%BB%E5%AD%90%E5%9E%83%E5%9C%BE%E5%B4%97.

[9] Wang Z., Zhang B., Guan D. (2016). Take Responsibility for Electronic-Waste Disposal. Nature.

[10] Parties to the Basel Convention on the Control of Transboundary Movements of Hazardous Wastes and their Disposal. http://www.basel.int/Countries/StatusofRatifications/PartiesSignatories/tabid/4499/Default.aspx#CN5.

[11] Cap. 354 Waste Disposal Ordinance. https://www.elegislation.gov.hk/hk/cap354!en-zh-Hant-HK?INDEX_CS=N.

[12] Klijn E.H., Walter J.M.K., Klijn E.H., Koppenjan J.F.M. (1997). Policy Networks: An Overview. Managing Complex Networks: Strategies for Public Sector.

[13] Bevir M. (2009). Key Concepts in Governance.

[14] Mizruchi M.S., Galaskiewicz J. (1993). Networks of Internorganisational Relations. Sociol. Methods Res..

[15] Carlsson L. (2000). Policy Networks as Collective Action. Policy Stud. J..

[16] Renckens S. (2008). Yes, We Will! Voluntarism in US E-Waste Governance. Rev. Eur. Community Int. Environ. Law.

[17] Wagner T.P. (2009). Shared Responsibility for Managing Electronic Waste: A Case Study of Maine, USA. Waste Manag..

[18] Bisschop L., Wyatt T. (2016). How E-Waste Challenges Environmental Governance. Hazardous Waste and Pollution.

[19] Chung S., Lau K., Zhang C. (2011). Generation of and Control Measures for, E-Waste in Hong Kong. Waste Manag..

[20] Yu J., Williams E., Ju M., Shao C. (2010). Managing E-Waste in China: Policies, Pilot Projects and Alternative Approaches. Resour. Conserv. Recycl..

[21] Padiila Y.C., Daigle L.E. (1998). Inter-Agency Collaboration in an International Setting. Adm. Soc. Work.

[22] Knowing Who I Am and What I Know: Developing New Versions of Professional Knowledge in Integrated Service Settings. http://www.leeds.ac.uk/educol/documents/00001877.htm.

[23] Hills P., Zhang L., Liu J. (1998). Transboundary Pollution Between Guangdong Province and Hong Kong: Threats to Water Quality in the Pearl River Estuary and Their Implications for Environmental Policy and Planning. J. Environ. Plan. Manag..

[24] One Country, Two Systems, One Smog Cross-Boundary Air Pollution Policy Challenges for Hong Kong and Guangdong. https://www.wilsoncenter.org/sites/default/files/3-feature_2.pdf.

[25] Hills P., Roberts P. (2001). Political Integration, Transboundary Pollution and Sustainability: Challenges for Environmental Policy in the Pearl River Delta Region. J. Environ. Plan. Manag..

[26] Lee Y. (2002). Tackling Cross-Border Environmental Problems in Hong Kong: Initial Responses and Institutional Constraints. China Q..

[27] Scam Recycling: E-Dumping on Asia by US Recycler, the E-Trash Transparency Project, Basel Action Network. http://wiki.ban.org/images/1/12/ScamRecyclingReport-web.pdf.

[28] E-Waste in China: A Country Report https://collections.unu.edu/eserv/UNU:1624/ewaste-in-china.pdf.

[29] Asian Network Workshop 2017 Updates on Waste Import and Export Control in Hong Kong SAR. https://www.google.com/url?sa=t&rct=j&q=&esrc=s&source=web&cd=1&ved=2ahUKEwixhfa175zeAhXBf7wKHUeaCbsQFjAAegQICRAC&url=https%3A%2F%2Fwww.env.go.jp%2Fen%2Frecycle%2Fasian_net%2FAnnual_Workshops%2F2017_PDF%2FS1_10_Hong_Kong_updated.pdf&usg=AOvVaw2dSqcrkQ0toRTzQ6l56fOg.

[30] Recovery of Waste Electrical and Electronic Equipment Recycling Programme. https://www.wastereduction.gov.hk/en/workplace/weee_intro.htm.

[31] Shunichi H., Khetriwal D., Ruediger K. (2016). Regional E-waste Monitor: East and Southeast Asia.

[32] Computer and Communication Products Recycling Programme. https://www.wastereduction.gov.hk/en/workplace/crp_intro.htm.

[33] Locations of Collection Point, Environmental Protection Department, The Government of the Hong Kong Special Administrative Region. https://www.wastereduction.gov.hk/en/quickaccess/vicinity.htm?collection_type=bin&material_type=all&district_id=0.

[34] Report on the Development of Renewable Resources Recycling Industry 2018. http://ltfzs.mofcom.gov.cn/article/ztzzn/an/201806/20180602757116.shtml.

[35] Eco Park, Hong Kong. www.Ecopark.com.hk.

[36] About EcoPark, EcoPark, Department of Environmental Protection. http://www.ecopark.com.hk/en/about.aspx.

[37] GEM Co. Ltd Company Profile. http://en.gem.com.cn/index.php/gongsijianjie/.

[38] Beijing Huanwei Company Profile. http://www.besg.com.cn/surroundings/CompanyProfile.html.

[39] Hong Kong Blueprint for Sustainable Use of Resources 2013–2022, Environment Bureau. https://www.enb.gov.hk/sites/default/files/pdf/WastePlan-E.pdf.

[40] Producers Responsibility Schemes. https://www.epd.gov.hk/epd/english/environmentinhk/waste/pro_responsibility/index.html.

[41] Control on Import & Export of Waste. https://www.epd.gov.hk/epd/english/environmentinhk/waste/guide_ref/guide_wiec_faq.html.

[42] The Hong Kong Collector/Recycler Directory. https://www.wastereduction.gov.hk/en/quickaccess/vicinity.htm?collection_type=collector&material_type=all&district_id=0.

[43] Category for Electrical and Electronic Waste, Pollution Control and Prevention. http://www.mee.gov.cn/hjzli/gtfwgl/dzdcplfqw/.

[44] Detomasi D.A. (2007). The multinational corporation and global governance: Modelling Global Public Policy Networks. J. Bus. Ethics.

